# Total IgE Distribution in Food Allergy Suspected Patients in Republic of Macedonia (2001-2011)

**DOI:** 10.3889/oamjms.2015.045

**Published:** 2015-04-19

**Authors:** Slavica Hristomanova Mitkovska, Dejan Trajkov, Aleksandar Petlichkovski, Olivija Efinska-Mladenovska, Mirko Spiroski

**Affiliations:** *Institute of Immunobiology and Human Genetics, Faculty of Medicine, Ss Cyril and Methodius University of Skopje, Skopje, Republic of Macedonia*

**Keywords:** IgE-mediated food allergy, total IgE, specific IgE antibodies to allergens from foods of plant origin, IgE antibodies to allergens from foods of animal origin, Republic of Macedonia

## Abstract

**BACKGROUND:**

IgE may be considered the hallmark of allergic disorders. It is easily detected in serum and can be measured as total IgE and as allergen-specific IgE. In fact, the serum IgE assay is used to diagnose an allergy.

**AIM:**

The aim of this study is to evaluate, investigate and present the distribution of total serum IgE levels, determined with UniCap system, in food-allergy suspected patients in a Republic of Macedonia.

**MATERIAL AND METHODS:**

In this study we analyzed retrospectively 8898 consecutive patients that were admitted for allergy testing at the Institute of Immunobiology and Human Genetics during the ten year period between 01.01.2001 and 01.01.2011. Total IgE levels in patient sera were detected with the in vitro system UniCAP100 (Pharmacia, Uppsala, Sweden).

**RESULTS:**

When we analyzed the number of patients according to the total IgE groups, we noted that most of the patients have normal levels of total IgE in serum. However, we also discovered a group of patients with elevated levels of total IgE that are greater than 200 kU/L. The average concentration of total serum IgE is higher in women in the age group 6 (6-7 years), followed by a steep decrease in the age group 9 (9-10 years), and after that the average concentrations of total IgE were mostly constant with the exception of a partial increase in the age group 21 (65-69 years). For men, the average serum concentrations of total IgE were highest in the age group of 6 (6-7 years), which was significantly higher than the average concentrations of total IgE in all other age groups.

**CONCLUSION:**

The large number of enrolled patients, a particular strength of this study, revealed that average concentrations of total IgE in men are higher than in women and that total IgE did not decrease with age. On the contrary, increased total IgE levels were found in patients aged 65 and 69 of both genders. We continue our work with analyses of the specific IgE antibodies values toward food and the correlation with total IgE values.

## Introduction

The prevalence of food allergies is rising worldwide. In Asia, it has been reported that 4-5% of school age children in Singapore have a food allergy [1]. Australia has reported a growth of 350% in admissions episodes of anaphylaxis associated with food between 1994 and 2005, and most of them were in the age group of 0-4 years [2]. Increasing incidence of food allergy have been reported in the UK [3], USA [4] and Australia [5]. The term “food allergy” refers to a patient’s immune response directed towards food [6]. In 2010 this term was defined as: “an adverse health effect arising from a specific immune response that occurs reproducibly on exposure to a given food” [7]. This definition includes immune responses that involve IgE binding to the allergen and are referred to as IgE-mediated, non-IgE-mediated or represent combinations of both [8]. Therefore, IgE may be considered the hallmark of allergic disorders. It is easily detected in serum and can be measured as total IgE and as allergen-specific IgE. In fact, the serum IgE assay is used to diagnose an allergy.

IgE-mediated reactions are characterized by the acute onset of symptoms, usually within two hours after intake or exposure to food and symptoms frequently involve abnormalities of the skin, gastrointestinal tract and/or the respiratory tract. Even though any food may bear the potential for mediating an allergic reaction and more than 170 foods have been reported to cause IgE-mediated immune responses, it is a rather small number of foods that have been found to cause the majority of allergic reactions. They are described in the literature as “major food allergens’’, and include: peanuts, nuts, eggs, milk, fish, crabs, shellfish, wheat and soy [7]. Globally, the prevalence of food allergies is on the rise and increases in incidence are observed in almost all countries. Food allergy prevalence varies in a manner that is dependent on the respective culture and population.

The aim of this study is to evaluate, investigate and present the distribution of total serum IgE levels, determined with UniCap system, in food-allergy suspected patients addressed to Institute of Immunobiology and Human Genetics in a Republic of Macedonia within a 10 year period (2001-2011).

## Materials and Methods

### Subjects

**Figure 1:**
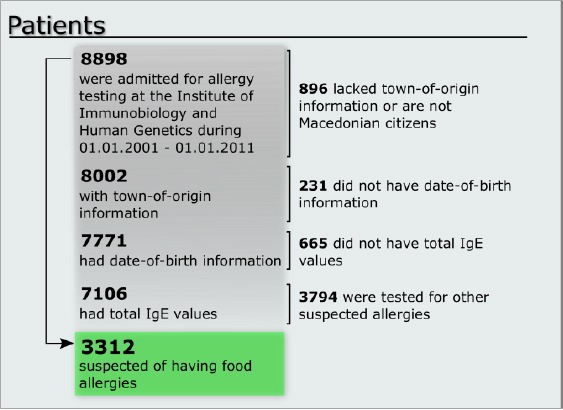
*Patient selection algorithm*.

In this study we analyzed retrospectively 8898 consecutive patients that were admitted for allergy testing at the Institute of Immunobiology and Human Genetics during the ten year period between 01.01.2001 and 01.01.2011. First, the patients were subjected to a selection algorithm, whose inclusion and exclusion criteria are shown in Figure 1.

After the implementation of the inclusion and exclusion criteria the analyses were conducted on the remaining 3312 patients for which we have accurate information regarding their origin, age, values for total serum IgE and values for specific IgE antibodies directed to food allergens. 1407 patients were women and 1905 men, aged between 1 month and 83 years. For the data analysis patients were organized into age groups as outlined in Table 1.

**Table 1:** Patients age groups as implemented.

Group	Age [years]	Group	Age [years]
0	0 - 1	13	25 - 29
1	1 - 2	14	30 - 34
2	2 - 3	15	35 - 39
3	3 - 4	16	40 - 44
4	4 - 5	17	45 - 49
5	5 - 6	18	50 - 54
6	6 - 7	19	55 - 59
7	7 - 8	20	60 - 64
8	8 - 9	21	65 - 69
9	9 - 10	22	70 - 74
10	10 - 14	23	75 - 79
11	15 - 19	24	80 - 84
12	20 - 24	25	85 - 90

### Methods

Total IgE levels in patient sera were detected with the *in vitro* system UniCAP100 (Pharmacia, Uppsala, Sweden) [9]. Anti-IgE covalently coupled to the ImmunoCAP vessel reacts with the total IgE in the patient sample. After washing, enzyme-labeled antibodies against IgE were added to form a complex and the bound complex was then incubated with a developing agent. After stopping the reaction, the fluorescence of the sample was measured. The fluorescence intensity was directly proportional to the IgE concentration in the sample. To evaluate the test results, the response of the respective patient sample was compared directly to that of the calibrators [10].

Serum concentrations of total IgE are associated with age. They increase during childhood and around 10 years of age reach levels that are maintained throughout life. Table 2 shows the normal total IgE values for the respective age in the Republic of Macedonia [11].

**Table 2:** Normal total IgE levels according to age

Age	Geometric mean (kU/L)	+ 1 SD (kU/L)	Age	Geometric mean (kU/L)	+ 1 SD (kU/L)
	**Weeks**			**Years**	
6	0.6	2.3	2	5.7	23
	**Months**		3	8.0	32
3	1.0	4.1	4	10	40
6	1.8	7.3	5	12	48
9	2.6	10	6	14	56
12	3.2	13	7	16	63
			8	18	71
			9	20	78
			10	22	85

### Statistical analyses

Results were grouped by sex, town of origin and age. The statistical analysis of the various frequency distributions and correlations were made with SPSS 20.0. Since the total IgE frequencies deviated from the normal distribution, analogue non-parametric tests independent of the distribution were applied. To lower the peak of the graphs for the total IgE levels, we applied logarithmic transformations to the dataset after which the same non-parametric tests were repeated. Descriptive statistics are expressed as means and standard error of the mean. The non-parametric Kruskal-Wallis rank test and Welch’s ANOVA test were performed. Values of *P* ≤ 0.05 were considered to indicate statistical significance. Differences in total IgE levels between men and women were investigated through application of the t-test.

## Results

**Figure 2:**
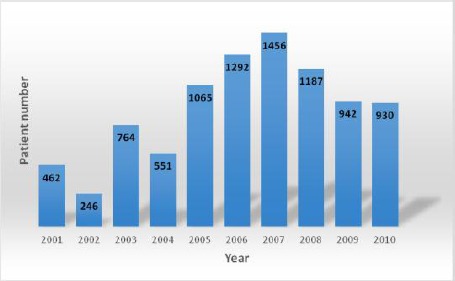
*Number of patients admitted to the Institute of Immunobiology and Human Genetics organized by year*.

First, we analyzed the frequency of patients addressed to the Institute of Immunobiology and Human Genetics for allergy testing (Figure 2) and we noticed an overall increase in patient number between 2004 and 2007, after which the total patient number per year remained constant.

**Figure 3:**
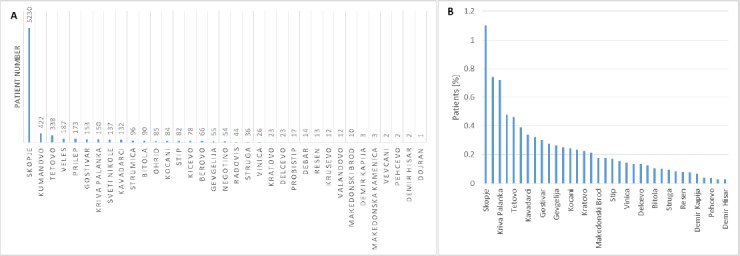
*A) Number of patients admitted in ten year period organized by town; B) Percentage of admitted patients normalized to the number of residents in the respective town*.

In further analyses, we ranked the admitted patients according to their town of origin (Figure 3A). Figure 3B shows the percentage of admitted patients in terms of the number of inhabitants of their town of origin. We used estimates for the number of inhabitants in 2004, which are based on the last Macedonian census conducted in 2002. We used these estimated data for the analyses because 2004 is approximately in the middle of the period that we investigated. We noticed significant differences in the distribution of the patients according to the respective town of origin.

**Figure 4:**
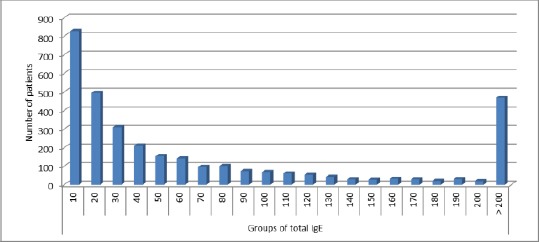
*Distribution of patients in groups for the concentration of total IgE*.

Figure 4 shows patient number per total IgE group and revealed that most patients had normal total IgE levels.

The number of admitted patients did not correlate with the total IgE levels per group. The total IgE concentration showed an increase in age group 6, followed by a decrease in group 7 and an increase in age group 8. The concentrations of total IgE remain constant over the next groups. However, in group 21 (patients aged 65 to 70 years) we observed an increase in total IgE levels.

The Kruskal-Wallis test result was: H (24) = 466.944 p <0.01 <0.05; which means that there is a statistically significant difference in the values of total IgE among specific age groups. Therefore, the null hypothesis (there is no statistically significant difference between the different age groups for the total IgE) had to be rejected in favor of the alternative hypothesis.

The results from the ANOVA test were F (24.3287) = 8.834, p < 0.001, which means that there is a statistically significant difference between the arithmetic means of total IgE between the different age groups. But since the Levenov test showed a difference in the variances, we could not use the basic ANOVA test (and there is no Gaussian distribution). Therefore, the Welch and Brown-Forsythe test were performed. They showed a statistically significant difference (in both cases) between the variances, and the result W (24) = 7.797, p < 0.001 and BF (24) = 5.147, p < 0.002.

Further we examined the concentration of total IgE in men and women (Figure 6A and 6B), as well as the natural logarithm in both categories (Figure 6C and 8D).

**Figure 5:**
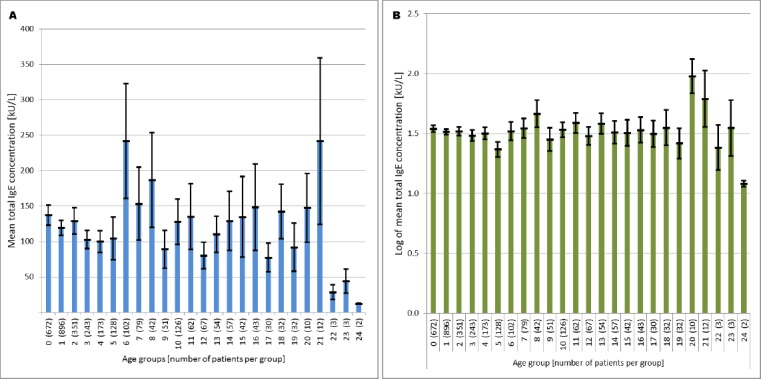
*A) Distribution of total IgE between the age groups of patients; B) Distribution of log total IgE between age groups of patients; Serum IgE concentrations are shown as columns and standard error of mean values as line*.

**Figure 6:**
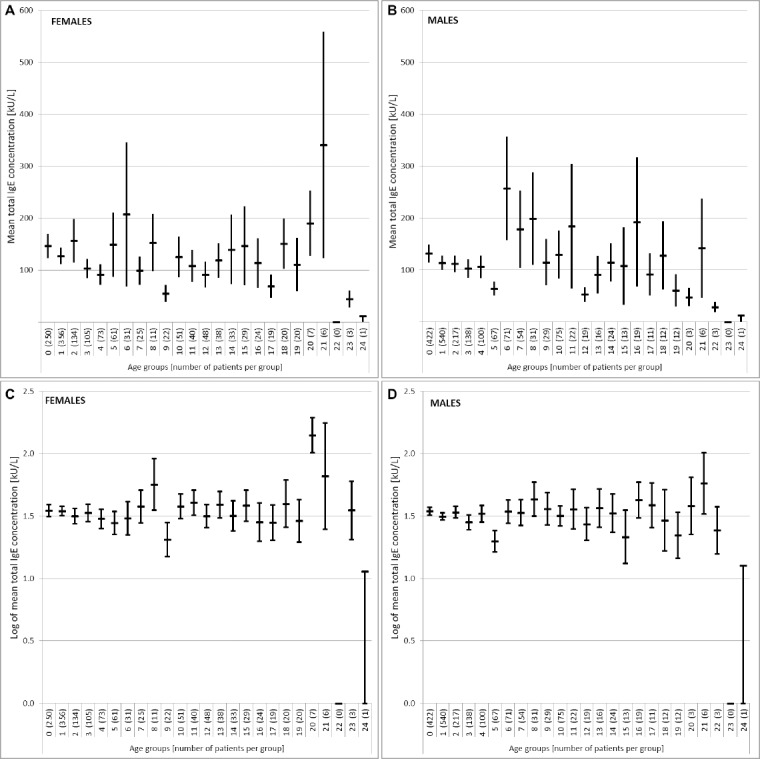
*The distribution of the average concentration of total IgE and standard error among females (A) and males (B); Distribution of the logarithm of the average concentration of total IgE and standard error among females (C) and males (D)*.

The average concentration of total serum IgE is higher in women in the age group 6, followed by a steep decrease in the age group 9, and after that the average concentrations of total IgE were mostly constant with the exception of a partial increase in the age group 21. For men, the average serum concentrations of total IgE were highest in the age group of 6, which was significantly higher than the average concentrations of total IgE in all other age groups. In both sexes we saw repeated increase in the concentration of total IgE in the age group 21, which is lower in men compared with women in the same age group.

A t-test was performed to determine the significance of the difference of total IgE in both sexes. The obtained results are shown in Table 3 or p < 0.01, t (3271) = 3732; p = 0.001 <0.01 <0.05, which means that there is a statistically significant difference between total IgE in men and women.

**Table 3:** Values of total IgE and Log of total IgE in men and in women.

		Male	Female

Total IgE	Arithmetic mean	145.24	101.12

	SD	372.61	306.75

	Geometric mean	37.35	27.65

LOG Total IgE	Arithmetic mean	1.57	1.44

	SD	0.71	0.67

	Geometric mean	1.38	1.26

Arithmetic mean from the age (in years)		5.90	9.67

**Table 4:** T-test for total IgE distribution between men and women.

	Levene’s Test for Equality of Variances		t-test for Equality of Means						
	F	Sig.	t	df	Sig. (2-tailed)	Mean Difference	Std. Error Difference	95% Confidence Interval of the Difference	
								Lower	Upper
Equal variances assumed	23.78	.00	3.63	3310.00	.00	44.11	12.17	20.26	67.97
Equal variances not assumed			3.73	3271.32	.00	44.11	11.82	20.93	67.29

## Discussion

Food allergy usually manifests in early childhood at one year of age. In children, over 80% of the reactions to food result from milk, eggs, soy, wheat, peanuts, nuts, while in adults the most due to peanuts, nuts, shellfish and fish [12].

Analyses of admitted patients at the Institute of Immunobiology and Human Genetics in the period 01.01.2001 until 01.01.2011 (Figure 2) showed that there is a continuous increase in the number of patients admitted for allergy testing. The patient number remained constant in the last two years. The overall increase is consistent with the global rise in allergies.

According to a number of studies the heterogeneity of the allergies depends on the geographical position [13-16]. Figure 3 shows the distribution of all admitted patients in a ten year period according to the town of origin. The figure shows that the majority of patients are from Skopje, which is understandable because it is the largest city in the country, with Kumanovo and Tetovo following in second and third place and so on. When we expressed the admitted patients in terms of the number of residents in the respective towns (Figure 3B) Kriva Palanka replaced Kumanovo in the second place. The percentage of patients sent for allergy-testing from all towns, except Skopje, is less than 1% and compared with other countries is very low.

According to another research in Republic of Macedonia from 2006, the most of the patients with hypersensitivity to food came from Prilep and Dojran [17]. However, that study is based only on surveys of self-reported hypersensitivity to food.

Differences in admitted patients can be explained by different awareness/unawareness of family doctors for the analyses performed at the Institute and the geographical distance of the towns. Our findings reveal a clear need for the establishing of new allergy testing laboratories in additional towns throughout Macedonia.

When we analyzed the number of patients (Figure 4) according to the total IgE groups, we noted that most of the patients have normal levels of total IgE in serum. However, we also discovered a group of patients with elevated levels of total IgE that are greater than 200 kU/L.

If we analyze the frequency of patients in each age group (Figure 5), we can see a significant increase in the number of admitted patients between 1 and 2 years, and between 10 and 15 years of age. This can be explained by the fact that newborns have physiological immunodeficiency [18]. However, at birth they have immunoglobulin G from the mother, which crosses through the placenta, and of course there is the secretory IgA from the breast milk. These passively transferred antibodies can protect newborns until 18 months of age, although their response is usually short and with low-affinity [19, 20]. This is one of the reasons why most of the admitted patients are around two years of age. During adolescence, the human body is subject to a wide range of physical, physiological and immunological changes. These changes begin with and are mediated by different hormones. Depending on the gender the change begins at the age of 10 and 12 years [21].

Analyses of total IgE distribution by age group showed significant differences matching those published in literature [22]. In particular, the geometric mean for serum IgE was higher in men compared to women (Table 3). We noticed an increase in total IgE concentrations in the age group 6 and 21 in both genders. Still, the t-test revealed that there is a statistically significant difference between total IgE in men and women. However, while the average concentrations of total IgE in men are higher than in women, the total IgE concentration increase in women in the age group 21 is higher than in men. The literature data on gender differences in total IgE are inconclusive. Some authors noted that men have higher serum levels of total IgE then women [23, 24], while other authors did not observe differences in total IgE between the sexes [25-27]. Contrary to our results, some authors observed a higher increase in the concentrations of total IgE in older men only [28], while we determined a total IgE increase in both sexes of that age group.

From the presented results we can interpret that the values for total IgE did not decrease with aging. One possible explanation for this may be that during the aging there is damage the regulatory function of the immune system [29].

We also found different data in the literature for the question of whether the levels of total IgE decline over the life. Most authors have published a decrease of the levels of IgE with aging in both men and women [23, 25, 30], while other authors have described a decline in the concentration of total IgE with aging in women, but not in men [28].

In conclusion, the large number of enrolled patients, a particular strength of this study, revealed that average concentrations of total IgE in men are higher than in women and that total IgE did not decrease with age. On the contrary, increased total IgE levels were found in patients aged 65 and 69 of both genders. We continue our work with analyses of the specific IgE antibodies values toward food and the correlation with total IgE values.
